# Analysis of Rainfall Infiltration Law in Unsaturated Soil Slope

**DOI:** 10.1155/2014/567250

**Published:** 2014-01-30

**Authors:** Gui-rong Zhang, Ya-jun Qian, Zhang-chun Wang, Bo Zhao

**Affiliations:** ^1^Geotechnical Engineering Institute of Nanjing Hydraulic Research Institute, Nanjing 210024, China; ^2^State Key Laboratory of Hydrology-Water Resources and Hydraulic Engineering, Nanjing 210024, China

## Abstract

In the study of unsaturated soil slope stability under rainfall infiltration, it is worth continuing to explore how much rainfall infiltrates into the slope in a rain process, and the amount of rainfall infiltrating into slope is the important factor influencing the stability. Therefore, rainfall infiltration capacity is an important issue of unsaturated seepage analysis for slope. On the basis of previous studies, rainfall infiltration law of unsaturated soil slope is analyzed. Considering the characteristics of slope and rainfall, the key factors affecting rainfall infiltration of slope, including hydraulic properties, water storage capacity (*θ*_s_ - *θ*_r_), soil types, rainfall intensities, and antecedent and subsequent infiltration rates on unsaturated soil slope, are discussed by using theory analysis and numerical simulation technology. Based on critical factors changing, this paper presents three calculation models of rainfall infiltrability for unsaturated slope, including (1) infiltration model considering rainfall intensity; (2) effective rainfall model considering antecedent rainfall; (3) infiltration model considering comprehensive factors. Based on the technology of system response, the relationship of rainfall and infiltration is described, and the prototype of regression model of rainfall infiltration is given, in order to determine the amount of rain penetration during a rain process.

## 1. Preface

In the stability analysis of landslide, rainfall is a very important factor. A large amount of statistical data shows that most of landslides occur after a rainfall or during a rainfall. There exists a law that landslides increase with increased rainfall in a region. Such rain-induced landslide and slope failure are the most common ones in many countries such as Japan, Hong Kong, and Southeast Asia.

With the development of percolation theory of saturated and unsaturated soil, both at home and abroad, researchers have been increasingly aware that the occurrences of soil slopes have close relationship with soil unsaturated seepage in rainy season. The physical process of rainfall infiltration into ground and its seepage through unsaturated-saturated soils has been studied by hydrogeologists, soil scientists, and geotechnical researchers. Landslides of Three Gorges Reservoir are mainly clay fragments with gravel, and slide belts are mainly soft clay, silty clay with gravel, or the clasts of sandstone or mudstone. As to this type of landslide, main influence of the rainfall is rainfall infiltration changes seepage field of sliding body.

**Figure 1:**
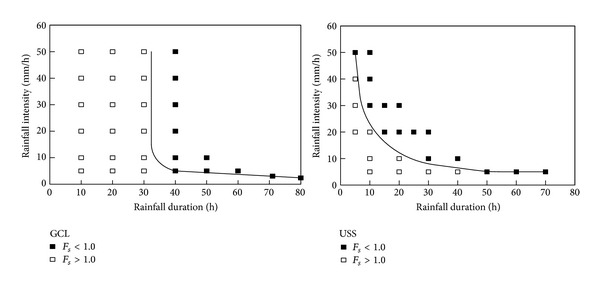
Effect of rainfall intensity and duration on slope stability [8]. (GCL: Glendale clayey loam, *k*_*s*_ = 1.5 × 10^−4^ cm/s; USS: uplands silty sand, *k*_*s*_ = 18.3 × 10^−4^ cm/s).

In order to analyze a rainfall infiltration process on slide, some factors can be considered, such as slope's characteristics, precipitation, evaporation, and other aspects. From the slope, the main factors include permeability of slope soil, slope, vegetation coverage, fissure distribution, and initial soil moisture content. To the rainfall, rainfall intensity, rainfall type, and rainfall duration have important influences on rainfall infiltration process. For the evaporation, evaporation intensity and duration can be considered as the crucial factors [1–6]. Landslides, with different geological and geo-morphological conditions, have different permeability and different discharge capacity; thus rainfall effects are different for different material composition of landslides. As in Hong Kong, most sliding body materials are noncohesive soil with good permeability, so slope occurrences have close relevance with high intensity and short-time rainfall. This result had been verified in the researches of Au [7] and Cai and Ugai [8] (Figure 1). In Singapore, sliding body materials are mainly two categories of residual soil, silty clay and sandy silt of hard plastic with poor permeability, so cumulative rainfall effect is very important for landslide occurrence. The article of Rahardjo et al. [9] is a good reflection of this view.

In the relevant studies on rainfall infiltration, especially in landslide research field, rainfall seepage is simplified in many articles, which is assumed to be a constant. For example, Fredlund [10] assumed the seepage to be 10% in slope infiltration, 100% for horizontal ground, and there existed difference between assumed values and actual infiltration. Therefore, during the study of rainfall effects on landslide, the amount of water infiltration in a rainfall process has important influences on negative pore-water pressure of the soil and thus on landslide stability, which requires further studies.

Ding and Liu [11] established a semiempirical model on rainfall infiltration and calculated model coefficients of infiltration recharge to groundwater for different soils, by using statistical analysis method. Wang et al. [12] calculated rainfall infiltration coefficient based on dynamic observation of groundwater and rainfall observation data, and Wang et al. thought that it was appropriate to calculate the coefficient using effective rainfall, and topsoil, groundwater level, and other factors should be fully considered. Guo [13] pointed that the coefficient of annual rainfall infiltration is an important hydrogeology parameter for groundwater recharge, and analyzed the coefficient by using groundwater level and rainfall data of typical years (2003, 2006, and 2007) in Cangzhou region.

Xie et al. [14, 15] developed the geohazard forecast research based on the coupling of regional geology-meteorology and put forward an effective rainfall model. This model overcame the defects of previous studies, which mainly considered the rainfall and rarely the geological factors, causing low reliability and accuracy in geologic disaster prediction. However, this research mainly started from the statistical point of view and had not yet fully considered the characteristics of landslides, and the applicability needed to be improved.

## 2. The Basic Law of Rainfall Infiltration in Slope Surface

### 2.1. The Process of Rainfall Infiltration

According to the force and motion characteristics of water, a seepage process can be divided into three stages: (1) Imbibition stage: this stage is mainly molecular force. Infiltration water is absorbed by soil particle and becomes pellicular water. In the dry soil, this stage is very obvious. When soil water content is larger than maximum molecular moisture, this stage disappears gradually. (2) Leakage stage: this stage is capillary force and gravity action. Infiltration water is downward for unsteady flow in soil pores and gradually fills soil pores until the pores are all filled and saturated. Generally, the two stages can be both named leakage stage. (3) Osmotic stage: when soil pores are all filled and saturated with water, the water is downward for steady flow under the gravity. On the whole, water seepage is the movement of unsaturated water, and infiltration is saturated flow. In actual penetration process, there is no obvious boundary between theses stages.

Rainfall infiltration is controlled by the infiltration rate of a landslide, which is defined as the actual water amount in a rainfall process through the unit area of surface in per unit time, and with speed dimension typically is expressed in *i* or *q*_*i*_. If the rainfall intensity is less than the minimum infiltrability of the topsoil, the infiltration rate is equal to rainfall intensity. In this case, if the rainfall intensity is assumed to be constant, then the infiltration rate will remain constant. With continuous precipitation, moisture content of topsoil increases gradually, until it reaches a stable value. When rainfall intensity is larger than infiltration ability of unsaturated topsoil, part precipitation transforms into runoff or seeper, and others seep into underground. There are two destinations for the water that penetrates into underground: part of the water is stored in soil pores above the underground water level, and the excess water beyond the soil's water retention just recharges the groundwater.

### 2.2. The Governing Equation of Rainfall Infiltration for Unsaturated Soil

The governing equation for 1D vertical infiltration in unsaturated soils is given by the following equation [16]:

(1) $$\begin{array}{c} \frac{\partial }{\partial z}\left[k\left(\psi \right)\frac{\partial \left(\psi +z\right)}{\partial z}\right]=\frac{\partial \theta }{\partial t}, \end{array}$$

where *ψ* = pore-water pressure head (negative for unsaturated flow); *θ* = volumetric water content of a soil; *t* = time; *z* = elevation head.

Srivastava and Yeh [17] assumed that the relationship of the water content of permeability (*k*) and that of volumetric water content (*θ*) to *ψ* can be both exponential:

(2) $$\begin{array}{c} \theta =\theta_{r}+\left(\theta_{s}-\theta_{r}\right)e^{\alpha \psi },\\ k=k_{s}e^{\alpha \psi }, \end{array}$$

where *θ*_*s*_ = saturated volumetric water content of the soil; *θ*_*r*_ = residual volumetric water content; *k*_*s*_ = saturated permeability; *α* = coefficient representing the desaturated rate of the SWCC. For many soils, these two functions can be used to approximate their hydraulic characteristics with the four parameters (i.e., *α*, *θ*_*s*_, *θ*_*r*_, and *k*_*s*_) over a wide range of negative pore-water pressures [18]. However, the two functions do not account for the air entry value of a SWCC, probably because of the ease of obtaining an analytical solution of (2). With these two functions, the Richards equation can be transformed to the following linear equation:

(3) $$\begin{array}{c} \frac{\partial^{2}k}{\partial z^{2}}+\alpha \frac{\partial k}{\partial z}=\frac{\alpha \left(\theta_{s}-\theta_{r}\right)}{k_{s}}\frac{\partial k}{\partial t}. \end{array}$$

**Figure 2:**
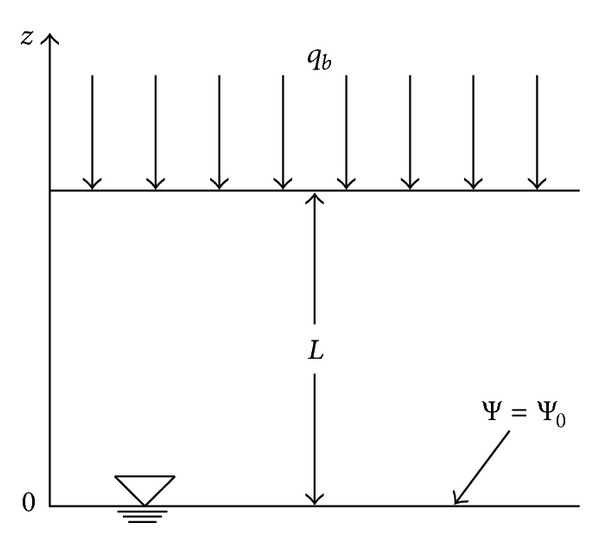
Homogeneous soil profile for calculation (based on [18]).

A one-dimensional transient infiltration problem generally involves one initial and two boundary conditions. In Srivastava and Yeh's study [17], the 1D infiltration problem in homogeneous soil is defined in Figure 2. The lower boundary is located at the stationary groundwater table, where negative pore-water pressure is equal to 0 (i.e., *ψ*_0_ = 0). Its boundary condition can be written as *k*|_*z*=0_ = *k*_*s*_.

The upper boundary at the ground surface is subjected to a rainfall infiltration (*q*), which is kept constant throughout the duration of rainfall considered. Its boundary condition can be expressed as

(4) $$\begin{array}{c} {\left(\frac{1}{\alpha }\frac{\partial k}{\partial z}+k\right)}_{z=L}=q. \end{array}$$

## 3. Main Factors Affecting the Rainfall Infiltration for Unsaturated Slope

Main factors, influencing rainfall infiltration of an unsaturated slope, include slope characteristics, precipitation, and evaporation. From the slope aspect, the main factors are slope seepage properties, slope gradient, vegetation coverage on slope surface, fissure distribution, and soil capillary water. From precipitation, rainfall intensities, rainfall types, and rainfall duration are the primary elements. To evaporation, the key factors are surface evaporation intensities and duration.

To precipitation and evaporation, they affect slope stability by mainly changing water distribution in slope, and they are external factors. The properties of slope itself are the internal factors, and the key is permeability of rock and soil.

### 3.1. Hydraulic Properties of Unsaturated Soil Slope

Similar to the flow through a saturated soil, water flow through an unsaturated soil is generally governed by Darcy's law. However, comparing the water flow in an unsaturated soil with the saturated flow, two major differences stand out: (1) there exits a storage term which represents the variation of water content with matric suction; (2) water permeability coefficient strongly depends on matric suction. It should be noted that no volume change in soil is considered during the infiltration process. The storage term in unsaturated flow is not a constant but depends on the suction in an unsaturated soil, and it can be characterized by the SWCC. Therefore, SWCC and water permeability coefficient are the most important hydraulic properties for unsaturated soils.

Two extreme cases of the infiltration problem can be first considered: water coefficient permeability tending to zero and that tending to infinity. At the former extreme there will be no infiltration of water into the soil stratum. At the latter extreme, water will infiltrate into the soil stratum easily but will immediately drain away through the boundaries. However, at intermediate values of water coefficient of permeability rainwater will infiltrate to a certain degree and will not entirely drain away. This implies that there exists a critical saturated permeability that may result in the greatest rainfall infiltration.

**Figure 3:**
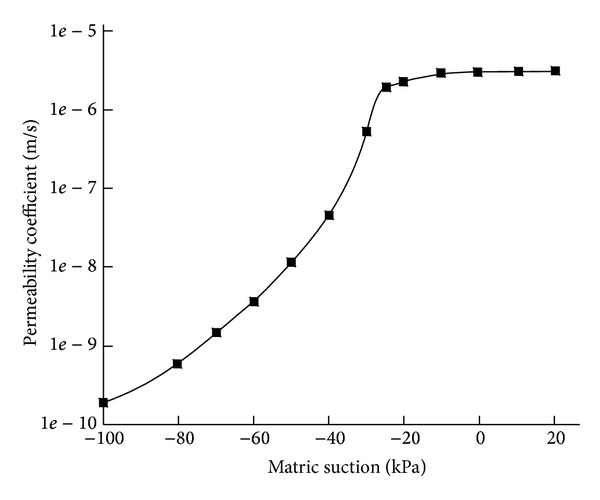
Curve of matric suction and permeability coefficient of cohesive soil.

**Figure 4:**
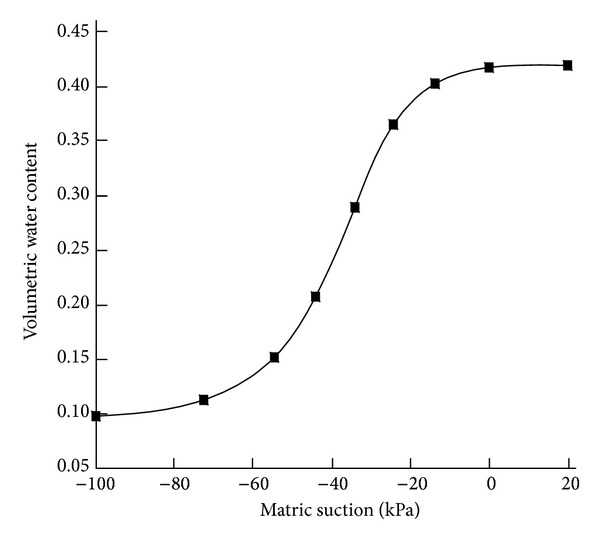
Soil water characteristic curve of cohesive soil.

In general, mass landslides occur with large rainfall intensity. From preceding analysis, in this case, the amount of water infiltrating into slope mainly depends on soil infiltration capacity, that is to say, permeability coefficient. For saturated soil, its permeability coefficient can be considered as a constant generally, and for unsaturated soil, its permeability coefficient changes with a large range; there are usually 3 to 5 orders of magnitude. Permeability coefficient of unsaturated soils is a function of volumetric water content (*K*(*θ*)), and volumetric water content is a function of pore-water pressure. Therefore, the water permeability coefficient is the indirect function of pore-water pressure. For cohesive soil, the typical relationship curves of matric suction and permeability coefficient and matrix suction and volumetric water content are shown in Figures 3 and 4.

From the graphs, with increase of volumetric water content, matric suction decreases rapidly. At the same time, permeability coefficient increases rapidly. When volumetric water content increases to saturation, matric suction decreases to zero; then permeability coefficient is equal to permeability coefficient of saturated soil.

### 3.2. Influence of Water Storage Capacity, (*θ*_*s*_ − *θ*_*r*_)

Water storage capacity of a soil, (*θ*_*s*_ − *θ*_*r*_), is equal to the difference between saturated and residual volumetric water content. It is a measurement of the maximum amount of water that can be absorbed or desorbed by capillary action. It is somewhat different from the soil's water retention ability. Generally, the value of (*θ*_*s*_ − *θ*_*r*_) increases with pore sizes. To some extent, it is also related to the void ratio.

### 3.3. Influence of Rock and Soil

**Figure 5:**
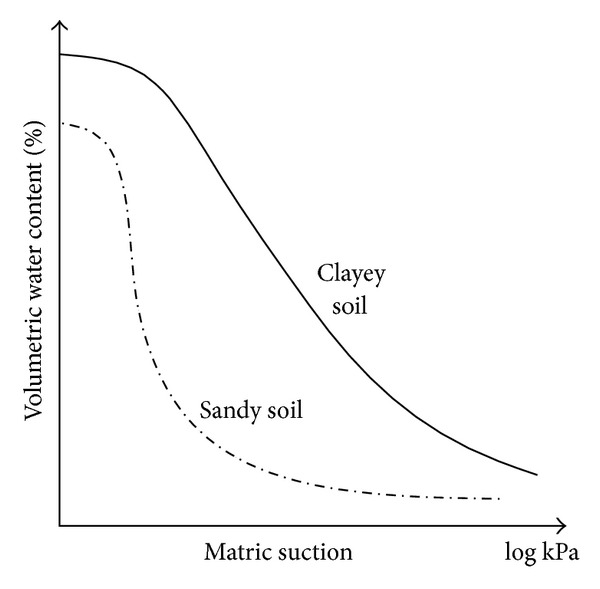
Soil water characteristic curves for different soils.

Figure 5 illustrates the general trend of SWCCs for two different types of soil. It can be seen that the shape and magnitude of the SWCC depend on soil types. In general, the greater the clay content, the larger the air-entry value, the greater the water retention ability at a given matric suction, and the more gradual the slope of the curve. In a sandy soil, most of the pores are relatively large, and once these larger pores are emptied at a given negative pore-water pressure, only a small amount of water remains. In a clayey soil, the pore size is relatively small and the distribution of pores is more uniform, with more water being adsorbed, so that the increase in matric suction causes more gradual decrease in water content.

### 3.4. Influence of Rainfall Intensity of Unsaturated Soil Slope

According to much research on this respect, the continuous rainfall, whose intensity does not exceed the infiltration rate, is the most favorable for rainfall infiltration. The input-regulating element calculates the actual infiltration rate into the slope considering the rainfall intensity and infiltration capacity, according to the following equations:

(5) q(t)=R(t),y(t)=0, R(t)<f(t),q(t)=f(t),y(t)=R(t)−f(t), R(t)>f(t),

where *R*(*t*) = rainfall intensity; *f*(*t*) = infiltration capacity; *q*(*t*) = infiltration rate of into upper soil layer; *y*(*t*) = intensity of rainfall excess.

### 3.5. Influences of Antecedent and Subsequent Infiltration Rate

Rainfall infiltration rate depends on rainfall intensity as well as the water permeability coefficient and the hydraulic gradient of topsoil. Both the water permeability coefficient and hydraulic gradient vary throughout the infiltration process due to the variations of negative pore-water pressure in the soil. The initial pore-water distribution is an essential input for transient flow analyses in unsaturated soils. Previous studies indicate that the antecedent infiltration rate not only has a significant influence on the initial pore-water distribution and hence on the pore-water pressure redistribution as a result of subsequent rainfall infiltration, but also indirectly results in a change of the water permeability coefficient of the soil [19].

Variations in subsequent rainfall infiltration rate are often restricted by the hydraulic properties of the soil and ground conditions, and rainfall infiltration in initially unsaturated soils results in an increase in the water permeability coefficient due to an increase of water content, and it simultaneously leads to a decrease of hydraulic gradient due to a decrease of negative pore-water pressure. If both the antecedent and subsequent rainfall infiltration rates are highest, the change in negative pore-water pressure after the rainfall will be the lowest. Therefore, it can be imagined that the negative pore-water pressure in the soil may be greatly reduced if a prolonged antecedent rainfall is combined with a heavy subsequent rainstorm.

## 4. Discussions on the Calculation Models of Rainfall Infiltration Capacity for Unsaturated Slope

### 4.1. Infiltration Model considering Rainfall Intensity

An infiltration model is used to estimate the rainfall excess in runoff analysis and is important for accurate simulation of a runoff hydrograph. Many infiltration models must accommodate rainfall intensities that are sometimes higher and sometimes lower than the infiltration capacity. The infiltration capacity depends on the moisture content in the upper soft layer which in turn depends on the rainfall intensity. Therefore, the infiltration capacity varies when the rainfall intensity varies.

**Figure 6:**
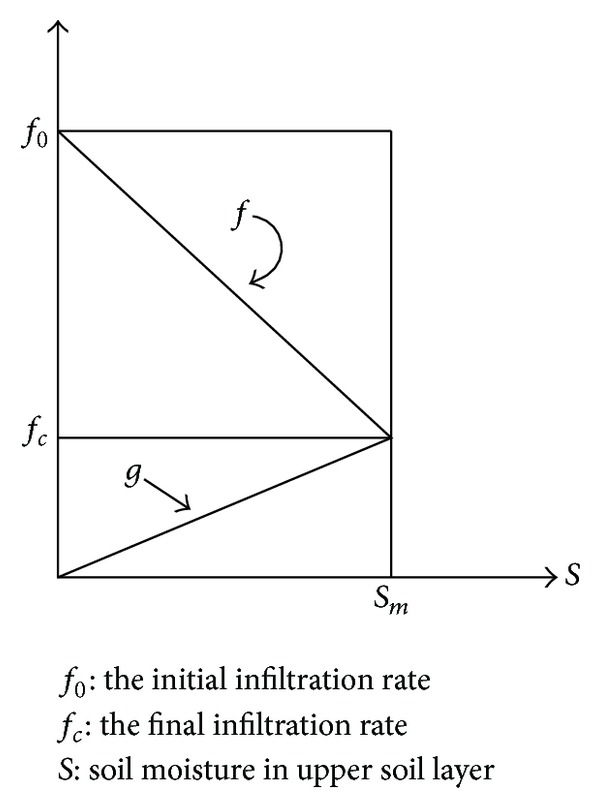
Relationship between infiltration capacity and soil moisture.

Only a few infiltration models take rainfall intensities into consideration; however, most of them are described using equations with a physical basis. The physical model is a theoretical one for unsaturated soil moisture movement, but the model treatment in terms of computation and modeling of the rainfall-runoff process may be more complex than the conceptual model when considering the rainfall intensity. The infiltration model proposed recently by Fujimura and Ando is a fairly simple conceptual model [20]. The relationship between the soil moisture content in the upper soil layer and the infiltration capacity is assumed to be specified by a decreasing linear relationship and is expressed as (6). At the same time, the percolation from the upper soil layer is assumed to be specified by an increasing linear relationship between the soil moisture content in the upper soil layer and the infiltration capacity and is expressed as (7). Both relationships are shown in Figure 6:

(6) $$\begin{array}{c} f\left(t\right)=f_{0}-\frac{\left(f_{0}-f_{c}\right)}{S_{m}}\times S\left(t\right), \end{array}$$

(7) $$\begin{array}{c} g\left(t\right)=\frac{f_{c}}{S_{m}}\times S\left(t\right). \end{array}$$

The equation of continuity for storage in the slope is expressed by subtracting the percolation rate from the actual infiltration rate as follows:

(8) $$\begin{array}{c} \frac{dS\left(t\right)}{dt}=q\left(t\right)-g\left(t\right)\;\left(0\leq S\left(t\right)<S_{m}\right), \end{array}$$

where *R*(*t*) = rainfall intensity; *f*(*t*) = infiltration capacity; *q*(*t*) = infiltration rate into upper soil layer; *y*(*t*) = intensity of rainfall excess; *S*(*t*) = soil moisture in upper soil layer; *S*_*m*_ = maximum value of soil moisture; *g*(*t*) = rate of percolation from upper soil layer.

### 4.2. Effective Rainfall Model considering the Antecedent Rainfall

Rainfall-induced landslides are the results of coworks of three basic rain variables (rainfall, rain intensity, and rain duration). Rain, inducing the occurrence or landslides reactivity, is generally the some rain with the maximum characteristic parameter, such as the greatest continuous rain, the longest continuous rain, the most strength continuous rain, or the greatest combination rain. In order to decide which rainfall has the closest correlation with landslide occurrence, that is to say, how to decide critical rainfall by using variables, Zhang et al. [21] developed the step regression analysis between numbers of landslide occurrences monthly by monthly and antecedent rain factors and calculated the correlation coefficients of landslide and rain factors.

Considering that one rain process not always induce landslide occurrences and only part rainfall of one rain process influences landslide, cumulative rainfall cannot be deemed as critical rainfall obviously. Hence, effective rainfall is recognized, which could be obtained by using this day's rainfall in some time multiplying effective rainfall coefficient. While deciding effective rainfall coefficient, it intends to adopt power exponent form in the following:

(9) $$\begin{array}{c} R_{c}=R_{0}+\alpha R_{1}+\alpha^{2}R_{2}+\cdots \alpha^{n}R_{n}, \end{array}$$

where *R*_*c*_ = effective rainfall; *R*_0_ = this day's rainfall; *R*_*n*_ = rainfall before n days; *α* = effective rainfall coefficient; *n* = the passing days. *α* is acquired according to Stat. analysis of historic information.

### 4.3. Infiltration Model considering Comprehensive Factors

The rainfall seepage is not only related with precipitation, characteristic of rock and soil, but related with groundwater level, preceding moisture content of slope, vegetation, and so on. Therefore, the crucial factors can be divided into external factors and intrinsic factors: (1) external factors: mainly refers to climatic conditions, such as rainfall intensities and duration, rainfall patterns, evaporation rate, groundwater level, and initial water content and (2) intrinsic factors: soil hydraulic properties, including water retention characteristic and water coefficient of permeability, slope gradient, and vegetation.

During the rainfall infiltration of landslide, the rainfall seepages to the slope surface firstly, then it is gradually infiltrates into deep slope with rainfall duration, and the excess water recharges the groundwater finally. Initial water distribution in slope soil directly determines rainfall infiltration rate and soil water capacity, thus affecting final infiltration amount. According to the principle of soil water balance, soil moisture in the slope depends mainly on rainfall, evaporation, and infiltration, and soil initial moisture content is mainly controlled by the antecedent rainfall and evaporation before the calculation period. That is to say, rainfall infiltration is a function of the current and antecedent rainfall, evaporation, soil initial water content, and other factors. The regression model of rainfall infiltration can be expressed as follows:

(10) $$\begin{array}{c} R\left(t\right)=\sum_{i=0}^{m}aP\left(t-i\right)+\sum_{j=0}^{n}bE\left(t-j\right)+cM+d\psi_{0}+S,\\ \alpha =\frac{R\left(t\right)}{P\left(t-i\right)}, \end{array}$$

where *R*(*t*) = rainfall infiltration of *t* time; *α* = rainfall infiltration coefficient of a rainfall process; *i* = the time of antecedent rainfall affecting this period of rainfall infiltration, *i* < *t*; *P*(*t* − *i*) = rainfall of *t* − *i* time; *E*(*t* − *j*) = evaporation of *t* − *j* time; *M* =  groundwater level; *ψ*_0_ =  initial moisture content of slope soil; *a*, *b*, *c*, *d* are constants; *m*, *n* are the time length of prophase rainfall and evaporation, respectively, and due to the different effects of rainfall and evaporation on infiltration degree, *m*, *n* can take different values; *S* = the contribution of all intrinsic factors.

It is essential to obtain the appropriate values for the above constants by trial and error methods, according to observation data of long time series, and combined with test methods.

## 5. Conclusions

This paper presents a simplified approach for the analysis of landslides triggered by rainfalls. Some conclusions can be attained.

1. In order to analyze the rainfall infiltration process on landslide, some factors can be considered. Considering the characteristics of slope and rainfall, the key factors are divided into external factors and intrinsic factors.
2. The influences of hydraulic properties, water storage capacity (*θ*_*s*_ − *θ*_*r*_), soil types, rainfall intensities, and antecedent and subsequent infiltration rate on unsaturated soil slope are to be discussed, and they are regarded as the most important factors affecting the rainfall infiltration for unsaturated slope.
3. Based on external factors changing, this paper presents three calculation models of rainfall infiltrability for unsaturated slope, including (1) infiltration model considering rainfall intensity; (2) effective rainfall model considering the antecedent rainfall; (3) infiltration model considering comprehensive factors.
4. In order to determine the amount of rain penetration during a rainfall process, the relationship of rainfall and infiltration is described by the system response analysis, and the prototype of regression model of rainfall infiltration is given.
